# Breast density and mode of detection in relation to breast cancer specific survival: a cohort study

**DOI:** 10.1186/1471-2407-14-229

**Published:** 2014-03-28

**Authors:** Åsa Olsson, Hanna Sartor, Signe Borgquist, Sophia Zackrisson, Jonas Manjer

**Affiliations:** 1 Department of Surgery, Lund University, Skåne University Hospital, SE- 205 02 Malmö, Sweden; 2 Diagnostic Radiology, Lund University, Diagnostic Center for Imaging and Functional Medicine, Skåne University Hospital Malmö, Malmö, Sweden; 3 Department of Oncology, Lund University, Skåne University Hospital, Lund, Sweden; 4 Department of Plastic surgery, Lund University, Skåne University Hospital, Malmö, Sweden

## Abstract

**Background:**

The aim of this study was to examine breast density in relation to breast cancer specific survival and to assess if this potential association was modified by mode of detection. An additional aim was to study whether the established association between mode of detection and survival is modified by breast density.

**Methods:**

The study included 619 cases from a prospective cohort, The Malmö Diet and Cancer Study. Breast density estimated qualitatively, was analyzed in relation to breast cancer death, in non-symptomatic and symptomatic women, using Cox regression calculating hazard ratios (HR) with 95% confidence intervals. Adjustments were made in several steps for; diagnostic age, tumour size, axillary lymph node involvement, grade, hormone receptor status, body mass index (baseline), diagnostic period, use of hormone replacement therapy at diagnosis and mode of detection. Detection mode in relation to survival was analyzed stratified for breast density. Differences in HR following different adjustments were analyzed by Freedmans%.

**Results:**

After adjustment for age and other prognostic factors, women with dense, as compared to fatty breasts, had an increased risk of breast cancer death, HR 2.56:1.07-6.11, with a statistically significant trend over density categories, p = 0.04. In the stratified analysis, the effect was less pronounced in non-symptomatic women, HR 2.04:0.49-8.49 as compared to symptomatic, HR 3.40:1.06-10.90. In the unadjusted model, symptomatic women had a higher risk of breast cancer death, regardless of breast density. Analyzed by Freedmans%, age, tumour size, lymph nodes, grade, diagnostic period, ER and PgR explained 55.5% of the observed differences in mortality between non-symptomatic and symptomatic cases. Additional adjustment for breast density caused only a minor change.

**Conclusions:**

High breast density at diagnosis may be associated with decreased breast cancer survival. This association appears to be stronger in women with symptomatic cancers but breast density could not explain differences in survival according to detection mode.

## Background

High breast density is an independent risk factor for breast cancer [1] but also decreases the sensitivity [2-4] for tumour detection by mammography [2-5].

The concept of breast density is based on the radiological appearance of the breast parenchyma and denser breasts have a higher proportion of epithelial and connective tissue in relation to fat, while non-dense breasts are richer in fat [6,7]. Breast density decreases after menopause [8] and with increasing body mass index (BMI) [9-11]. It has also been related to hormonal factors such as menopausal status and use of hormone replacement therapy (HRT) [8,11,12], but the biological mechanism connecting breast density to breast cancer risk is not clearly understood.

In order to increase sensitivity, shorter screening intervals have been suggested for younger women and/or women with denser breasts [13]. However, the effect of such interventions regarding mortality, or the potential effect of breast density on survival *per se*, is not known.

Six studies, have reported on breast density in relation to breast cancer specific survival. Two of the studies found that women with dense breasts had a slightly impaired survival [5,14], one found a statistically significant better survival in women with dense breasts [4], and two studies found no association at all [15,16]. In one study, breast density was associated with poorer survival only in women not receiving radiotherapy [17]. Women with screening detected breast tumours have a better prognosis compared to women with clinically diagnosed breast cancer, despite adjustment for stage at diagnosis and other tumour characteristics [18-21]. The prognostic advantage associated with mammography screening could be less evident in women with denser breasts, given the lower mammographic sensitivity. If breast density has an independent effect on survival, breast density would affect outcome regardless of detection mode, and might explain part of the survival difference between women with non-symptomatic vs. symptomatic tumours.

The aim of this study was to examine breast density in relation to survival following breast cancer diagnosis, using breast cancer specific death as the endpoint and to assess if this potential association was modified by mode of detection. An additional aim was to examine whether the established association between mode of detection and survival is modified by breast density.

## Methods

### The Malmö Diet and Cancer Study

The Malmö Diet and Cancer Study (MDCS) is a population based, prospective cohort study inviting residents in Malmö, Sweden, born between 1923 and 1950. Between 1991–1996, 17 035 women were enrolled, corresponding to a participation rate of approximately 40%. The study includes questionnaires and interviews on diet, medications, socio-economy and life-style factors [22,23]. Blood samples and information on weight and height were collected at baseline, and BMI was calculated as kg/m^2^[22,23].

### Identification of breast cancer patients

Data on cancer events in the MDCS-population has been retrieved from the Swedish Cancer Registry and The Regional Tumour Registry for Southern Sweden. Until 31 Dec. 2007, 826 incident breast cancer cases were diagnosed. Women with prevalent breast cancer at baseline (n = 576) were excluded. Participants in the Malmö Diet and Cancer Study have all given written informed consent at baseline. Through subsequent advertisements, included women have been informed about planned additional analyses and about the possibilities of withdrawal. No new contacts have been taken with included women or their relatives for this particular study. The present study was approved by The Ethical Committee at Lund University (Dnr 652/2005 and Dnr 166/2007).

### Screening status

This study included women from the MDCS cohort, potentially exposed to mammography screening. The general screening service started in Malmö in 1990 and was, during the study period, inviting women 50–69 years of age but with an extension of the upper age-limit to 74 years during the last decade. Women were invited at 18 or 24 months intervals depending on parenchymal pattern, (the shorter interval for women with denser breasts) [24]. Since there was no information on the presence of breast implants, or the use of opportunistic screening among participants in the general screening program, we refer to this group as non-symptomatic cancers. Mammography, and opportunistic screening, has to some extent been available outside the general screening program. Out of the final study population (n = 619, see below), 30 women were considered as diagnosed by screening outside general screening and they were classified as non-symptomatic if clearly stated in the clinical notes that they were asymptomatic at the time of the diagnostic mammogram. No information on screening intervals was available for this group. The diagnostic ages in women with non-symptomatic cancers ranged from 48 to 81 years, which were the limits used to define the present study population. An interval cancer was defined as a symptomatic breast cancer, diagnosed clinically within 18 or 24 months, (depending on the planned screening interval), from a previously normal screening mammogram.

### Study population

Out of 826 incident breast cancer cases, 79 cases with cancer in situ were excluded as the primary objective of the present study was to investigate survival. Fifteen cases with bilateral tumours were excluded due to the difficulty to retrospectively evaluate the stage of these tumours. Women with unknown screening status (n = 19) and unknown breast density (n = 36) were also excluded. Finally, 65 women were excluded due to insufficient amounts of tumour tissue. A single woman could be excluded for several reasons. Adding the age criteria 48–81 years for non-symptomatic cancers, the final study population included 619 cases. Out of these 619 women, 350 were non-symptomatic, 177 were symptomatic and 87 were interval cancers. In another five symptomatic women diagnosed clinically, it was not possible to exclude the possibility of an interval cancer. These women were included as ”symptomatic” in analyses using two categories of detection mode (non-symptomatic/symptomatic) and as “unknown” in analyses using three categories (non-symptomatic/interval/symptomatic). The eighteen year period of inclusion was divided into three six-year categories to define diagnostic period.

### Follow-up

Information on cause of death and vital status was retrieved from the Swedish Causes of Death Registry, with last follow up 31 Dec. 2010 [25]. At the end of follow up, 76 women had died from breast cancer as underlying or contributing cause of death (mean age at death: 70.1 years; standard deviation (SD): 8.6). Forty-seven women had died from other causes (mean age at death: 73.9 years; SD: 6.6). Median follow-up from diagnosis to death, end of follow-up or emigration (2 women) was 7.8 years (range 0.5-19.1).

### Tumour and patient characteristics

Information on tumour size, axillary lymph node involvement (ALNI), type of surgery, planned adjuvant therapy, menopausal status and use of HRT at diagnosis was collected from medical journals, including pathology reports. Fifty-eight women had not been operated in the axilla and thus had missing information on ALNI. In most of these cases, an axillary dissection had been considered unnecessary at the pre-operative evaluation and they were classified as ALNI negative. All these women had a tumour size less than or equal to 20 mm, and were free from distant metastases at diagnosis. One woman with distant metastases at diagnosis, but registered as having negative lymph nodes was classified as “unknown” for ALNI. The study population included four women with distant metastases at diagnosis, one diagnosed with an interval cancer and the other three had symptomatic tumours. Three of these women had died from breast cancer at end of follow-up. Cases diagnosed from study start in 1991 and until 31 Dec 2004, were re-evaluated regarding tumour type according to the World Health Organization-classification [26], and assessed for tumour grade according to Elston and Ellis [27] by one senior pathologist [28]. For cases diagnosed 1 Jan 2005 to 31 Dec 2007, information on tumour grade was collected from the pathology reports.

Tissue micro arrays for immunohistochemical analyses were constructed as described previously in order to define hormone receptor status; oestrogen receptor α (ER) and progesterone receptor (PgR) [28]. In this study, ≤10% or >10% of positive nuclei defined negative and positive hormone receptor status, in accordance with clinically used limits [29].

### Breast density

Breast density was estimated qualitatively and reported by experienced breast radiologists at the initial evaluation of the diagnostic mammogram*.* In the assessment of women recalled from screening with suspicion of breast cancer, the screening mammogram (craniocaudal and mediolateral oblique views) was completed with as many views as needed, corresponding to a diagnostic mammography examination with at least three views. Thus, the assessment of breast density was done at the time of the diagnostic work-up and not at the screening readings. Breast density was measured using both breasts and all views, although when there was an apparent effect of the tumor on the surrounding tissue in terms of higher breast density, the contralateral view was used. When breast density differed between breasts, not related to the tumour, the breast with the highest breast density was used for final decision. Information on breast density was missing in about one third of cases, and these mammograms were retrospectively revised by one breast radiologist (SZ) and a trained, supervised resident in radiology (HS). In 36 women, no mammograms were possible to find for revision. At end of follow-up, 11out of these 36 women had died from breast cancer and they were excluded from the study. The mammograms at the institution were analogue up until 2003 and digital from 2004 and onwards. Routinely, during the last 30 years, a three category classification of breast density has been used: “fatty”, “moderate” or “dense”. This classification is a modification of the Breast Imaging Reporting and Data System (BI-RADS) where “fatty” corresponds to BI-RADS 1 (almost entirely fat), “moderate” to BI-RADS 2 + 3 (scattered fibroglandular densities; and heterogeneously dense) and “dense” to BI-RADS 4 (extremely dense) [30].

For the descriptive analysis of the study-population, the three density categories described above were used. In some stratified analyses, fatty breasts and moderately dense breast were combined and compared to dense breasts.

### Methods

Factors related to the ability to diagnose a tumour; age, use of HRT, menopausal status and breast density at diagnosis, BMI at baseline, diagnostic period and mode of detection were compared according to outcome, defined as alive at end of follow-up, dead from breast cancer (as cause of death or contributing cause of death), or dead from other causes. Vital status and cause of death were further investigated in relation to known prognostic factors and treatment; diagnostic age, tumour size, ALNI, tumour grade, ER, PgR, type of surgery, type of lymph node examination and planned adjuvant treatment.

Factors related to the ability to diagnose a tumour were also investigated in relation to breast density. Differences were tested with ANOVA for continuous variables, and the Chi-2 test for categorical variables. All tests were two-sided and a p-value <0.05 was considered significant.

Breast density was analysed in relation to subsequent breast cancer death using Cox proportional hazards analysis calculating hazard ratios (HR) with 95% confidence intervals (CI). Adjustments were first made for prognostic factors; age, diagnostic period, tumour size, ALNI, grade, ER and PgR (HR^2^). Additional adjustments included BMI and HRT (HR^3^). The correlation between diagnostic age and menopausal status was tested using tau-b, analysing diagnostic age as a categorical variable (ten-year categories) and showed a statistically significant correlation, 0.405, p = <0.001, which is why the adjustments did not include menopausal status. All analyses were performed separately in non-symptomatic and symptomatic cases. In a final model, analysing all subjects, adjustments were also made for detection mode (HR^4^), and interactions between detection mode and breast density were tested using an interaction term. Linear trends over density categories were calculated yielding two-sided p-values. The association between detection mode and breast cancer death was analysed by the same model, first adjusting for prognostic factors as above (HR^2^ and HR^3^), and finally, in the analysis of all subjects, also for breast density (fatty/moderate/dense). Interactions between detection mode and breast density were tested using an interaction term. Freedmans% [31] was used to determine the contribution of these adjustments to the survival difference between non-symptomatic and symptomatic cases. Freedmans% was defined as: 100(1- a/b); where b is the logarithm of the unadjusted HR, and a, the logarithm of the adjusted HR.

The proportional hazard assumption was tested using a log minus log curve, and for all analyses on survival, the assumption was met. Missing values were included as separate categories in all multivariate analyses, thus, the adjustments made did not affect the number of included cases. All analyses were repeated excluding the four women with distant metastasis at diagnosis and all analyses were also repeated using death from causes other than breast cancer as the event and adjusted separately for age, BMI, HRT and diagnostic period as these factors are likely to affect overall mortality. A sensitivity analysis was made to the un-stratified Cox analyses, by adding adjustment for one modality of adjuvant therapy at the time (planned radio-therapy yes/no, planned chemo-therapy yes/no, planned antihormonal treatment yes/no). SPSS 20.0 was used for all calculations.

## Results

Women who died from causes other than breast cancer were slightly older at baseline and at diagnosis as compared to women alive at follow-up or women who died from breast cancer, Table 1. Few cases were pre-menopausal at diagnosis. A BMI ≥ 30 was more common among women dead from other causes, as compared to other groups. Women who died from breast cancer had less often non-symptomatic tumours and were somewhat more likely to have dense breasts, Table 1.

**Table 1:** Vital status and factors potentially related to breast density and tumour detection

**Factors**	**Category**	**Alive (n = 496)**	**Dead breast cancer (n = 76)**	**Dead other cause (n = 47)**	**All (n = 619)**
		**Number (column percent)** ***Mean, SD in italics***			
Age at baseline	Years	*56.0 (6.6)*	*58.2 (7.8)*	*61.6 (6.9)*	*56.7 (7.0)*
Age at diagnosis	Years	*64.0 (7.3)*	*64.5 (8.7)*	*67.8 (7.6)*	*64.4 (7.5)*
HRT at diagnosis	No	169 (34.1)	17 (22.4)	11 (23.4)	197 (31.8)
	Yes	168 (33.9)	21 (27.6)	11 (23.4)	200 (32.3)
	Unknown	159 (32.0)	38 (50.0)	25 (53.2)	222 (35.9)
Menopausal status at diagnosis	Premenopausal	40 (8.1)	11 (14.5)	2 (4.3)	53 (8.6)
	Postmenopausal	446 (89.9)	65 (85.5)	42 (89.4)	553 (89.3)
	Unknown	10 (2.0)	0 (0.0)	3 (6.4)	13 (2.1)
BMI at baseline	<20	21 (4.2)	4 (5.3)	2 (4.3)	27 (4.4)
	20- < 25	242 (48.8)	33 (43.4)	18 (38.3)	293 (47.3)
	25- < 30	159 (32.1)	30 (39.5)	14 (29.8)	203 (32.8)
	≥30	74 (14.9)	9 (11.8)	13 (27.7)	96 (15.5)
Detection mode (three categories)	Non-symptomatic	301 (60.7)	27 (35.5)	22 (46.8)	350 (56.5)
	Interval	68 (13.7)	13 (17.1)	6 (12.8)	87 (14.1)
	Symptomatic	122 (24.6)	36 (47.4)	19 (40.4)	177 (28.6)
	Unknown*	5 (1.0)	0 (0.0)	0 (0.0)	5 (0.8)
Detection mode (symptomatic including interval cancer)	Non-symptomatic	301 (60.7)	27 (35.5)	22 (46.8)	350 (56.5)
	Symptomatic	195 (39.3)	49 (64.5)	25 (53.2)	269 (43.5)
Breast density	Fatty	73 (14.7)	7 (9.2)	13 (27.7)	93 (15.0)
	Moderate	254 (51.2)	37 (48.7)	21 (44.7)	312 (50.4)
	Dense	169 (34.1)	32 (42.1)	13 (27.7)	214 (34.6)
Diagnostic period	1991-1996	62 (12.5)	21 (27.6)	11(23.4)	94 (15.2)
	1997-2001	173 (34.9)	28 (36.8)	19 (40.4)	220 (35.6)
	2002-2007	261 (52.6)	27 (35.5)	17 (36.2)	305 (49.3)

*Unknown if interval or symptomatic cancer.

Women who died from breast cancer had larger tumours (>20 mm), tumours of higher grade (grade III) and were more often ALNI positive and ER- and PgR negative, as compared to women alive at follow-up, or women dead from other causes, Table 2. Extensive surgery with mastectomy and axillary lymph node dissection was also more common among women who had died from breast cancer, and this group had more often been planned for chemo- or radiotherapy, Table 2.

**Table 2:** Vital status, prognostic factors and treatment

**Factor**	**Category**	**Alive (n = 496)**	**Dead breast cancer (n = 76)**	**Dead other cause (n = 47)**	**All (n = 619)**
		**Number (column percent)**			
Tumour size (mm)	≤10	160 (32.3)	1 (1.3)	13 (27.7)	174 (28.1)
	>10- ≤20	225 (45.4)	31 (40.8)	17 (36.2)	273 (44.1)
	>20	109 (22.0)	44 (57.9)	17 (36.2)	170 (27.5)
	Unknown	2 (0.4)	0 (0.0)	0 (0.0)	2 (0.3)
Axillary lymph node status (ALNI)	Negative	371 (74.8)	28 (36.8)	37 (78.7)	436 (70.4)
	Positive	124 (25.0)	48 (63.2)	10 (21.3)	182 (29.4)
	Unknown	1 (0.2)	0 (0.0)	0 (0.0)	1 (0.2)
Nottingham grade	I	152 (30.6)	7 (9.2)	14 (29.8)	173 (27.9)
	II	249 (50.2)	27 (35.5)	20 (42.6)	296 (47.8)
	III	91 (18.3)	42 (55.3)	12 (25.5)	145 (23.4)
	Unknown	4 (0.8)	0 (0.0)	1 (2.1)	5 (0.8)
Estrogen receptor status (ER)	Negative	47 (9.5)	19 (25.0)	5 (10.6)	71 (11.5)
	Positive	394 (79.4)	53 (69.7)	40 (85.1)	487 (78.7)
	Unknown	55 (11.1)	4 (5.3)	2 (4.3)	61 (9.9)
Progesterone receptor status (PgR)	Negative	182 (36.7)	44 (57.9)	26 (55.3)	252 (40.7)
	Positive	201 (40.5)	22 (28.9)	14 (29.8)	237 (38.3)
	Unknown	113 (22.8)	10 (13.2)	7 (14.9)	130 (21.0)
Type of surgery	Mastectomy	182 (36.7)	48 (63.2)	19 (40.4)	249 (40.2)
	Sector-resection	311 (62.7)	28 (36.8)	28 (59.6)	367 (59.3)
	Unknown	3 (0.6)	0 (0.0)	0 (0.0)	3 (0.5)
Axillary dissection	No	48 (9.7)	1 (1.3)	7 (14.9)	56 (9.0)
	Yes	280 (56.5)	63 (82.9)	33 (70.2)	376 (60.7)
	Single node	2 (0.4)	0 (0.0)	0 (0.0)	2 (0.3)
	Sentinel node	162 (32.7)	11 (14.5)	6 (12.8)	179 (28.9)
	Unknown	4 (0.8)	1 (1.3)	1 (2.1)	6 (1.0)
Antihormonal treatment	No	245 (49.4)	33 (43.4)	28 (59.6)	306 (49.4)
	Yes	242 (48.8)	42 (55.3)	19 (40.4)	303 (48.9)
	Unknown	9 (1.8)	1 (1.3)	0 (0.0)	10 (1.6)
Chemotherapy	No	380 (76.6)	51 (67.1)	42 (89.4)	473 (76.4)
	Yes	57 (11.5)	23 (30.3)	3 (6.4)	83 (13.4)
	Unknown	59 (11.9)	2 (2.6)	2 (4.3)	63 (10.2)
Radiotherapy	No	172 (34.7)	27 (35.5)	23 (48.9)	222 (35.9)
	Yes	269 (54.2)	47 (61.8)	22 (46.8)	338 (54.6)
	Unknown	55 (11.1)	2 (2.6)	2 (4.3)	59 (9.5)

Women with dense breasts were younger, more often premenopausal, HRT users and more likely to have a BMI < 25, as compared to women with fatty breasts, Table 3. No differences were seen regarding breast density and detection mode.

**Table 3:** Potential determinants for breast density and tumour detection

**Factor**	**Category**	**Breast density**			
		**Fatty (n = 93)**	**Moderate (n = 312)**	**Dense (n = 214)**	
		**Number (column percent)** ***Mean, SD in italics***			p-value**
Age at baseline	Years	*60.1 (7.1)*	*56.8 (6.8)*	*55.1 (6.7)*	<0.0001
Age at diagnosis	Years	*67.4 (7.3)*	*65.2 (7.1)*	*62.0 (7.6)*	< 0.0001
HRT at diagnosis	No	25 (26.9)	126 (40.4)	46 (21.5)	<0.0001
	Yes	10 (10.8)	84 (26.9)	106 (49.5)	
	Unknown	58 (62.4)	102 (32.6)	62 (29.0)	
Menopausal status at diagnosis	Premenopausal	3 (3.2)	18 (5.8)	32 (15.0)	0.001
	Postmenopausal	89 (95.7)	287 (92.0)	177 (82.7)	
	Unknown	1 (1.1)	7 (2.2)	5 (2.3)	
BMI at baseline	<20	0 (0.0)	10 (3.2)	17 (7.9)	<0.0001
	20- < 25	29 (31.2)	135 (43.3)	129 (60.3)	
	25- < 30	27 (29.0)	123 (39.4)	53 (24.8)	
	≥30	37 (39.8)	44 (14.1)	15 (7.0)	
Detection mode (three categories)	Non-symptomatic	50 (53.8)	183 (58.7)	117 (54.7)	0.333
	Interval	10 (10.8)	43 (13.8)	34 (15.9)	
	Symptomatic	31 (33.3)	83 (26.6)	63 (29.4)	
	Unknown*	2 (2.2)	3 (1.0)	0 (0.0)	
Detection mode (symptomatic including interval cancers)	Non-symptomatic	50 (53.8)	183 (58.7)	117 (54.7)	0.559
	Symptomatic	43 (46.2)	129 (41.3)	97 (45.3)	

*Unknown if interval or symptomatic cancer.

**Continuous variables analysed with ANOVA and categorical variables with the Chi-2 test.

High breast density was positively associated with death from breast cancer, and the HRs increased further following adjustments. There was also a dose–response pattern with a statistically significant trend over density categories in the adjusted model, including all cases, Table 4. The association between breast density and death from breast cancer was stronger among symptomatic women as compared to non-symptomatic. The p-values for interaction with mode of detection were for moderately dense breasts 0.021, and for dense breasts 0.006. In Table 5, where mode of detection was analysed stratified for breast density (in two categories), the p-value for interaction between symptomatic tumours and dense breasts was 0.685. However, these analyses included few events and confidence intervals were wide.

**Table 4:** Breast density and subsequent breast cancer mortality in relation to mode of detection

	**Rate of breast cancer deaths**	**HR**^**1**^	**HR**^**2**^	**HR**^**3**^	**HR**^**4**^
**All**					
Fatty	7 of 93	1.00	1.00	1.00	1.00
Moderate	37 of 312	1.82 (0.81-4.08)	1.80 (0.79-4.10)	2.02 (0.87-4.67)	2.09 (0.90-4.85)
Dense	32 of 214	1.92 (0.85-4.35)	2.24 (0.97-5.13)	2.56 (1.07-6.11)	2.63 (1.10-6.30)
* p-value for trend*		*0.173*	*0.058*	*0.039*	*0.035*
**Non-symptomatic**					
Fatty	3 of 50	1.00	1.00	1.00	–
Moderate	13 of 183	1.37 (0.39-4.81)	1.30 (0.36-4.76)	1.40 (0.38-5.17)	–
Dense	11 of 117	1.47 (0.41-5.27)	1.66 (0.43-6.41)	2.04 (0.49-8.49)	–
* p-value for trend*		*0.586*	*0.427*	*0.293*	*–*
**Symptomatic**					
Fatty	4 of 43	1.00	1.00	1.00	–
Moderate	24 of 129	2.43 (0.84-7.02)	2.20 (0.74-6.51)	2.62 (0.86-7.98)	–
Dense	21 of 97	2.33 (0.80-6.80)	2.71 (0.91-8.06)	3.40 (1.06-10.90)	–
* p-value for trend*		*0.216*	*0.081*	*0.045*	*–*

HR^1^: Crude.

HR^2^: Adjusted for diagnostic age (continuous), tumour size (mm), ALNI, grade, ER PgR and diagnostic period.

HR^3^ Adjusted for same factors as HR^2^ but also for HRT at diagnosis, BMI at baseline (continuous) and diagnostic period.

HR^4^: Adjusted for the same factors as HR^3^ but also for mode of detection (non-symptomatic or symptomatic).

**Table 5:** Mode of detection and subsequent breast cancer mortality in relation to breast density

	**Rate of breast cancer deaths**	**HR**^**1**^	**HR**^**2**^	**HR**^**3**^	**HR**^**4**^
**All**					
Non-symptomatic	27 of 350	1.00	1.00	1.00	1.00
Symptomatic	49 of 269	2.68 (1.67-4.28)	1.55 (0.95-2.53)	1.52 (0.92-2.50)	1.56 (0.94-2.58)
Freedmans%*			*55.5*	*57.5*	*54.9*
**Fatty/moderate**					
Non-symptomatic	16 of 233	1.00	1.00	1.00	–
Symptomatic	28 of 172	2.63 (1.42-4.87)	1.48 (0.76-2.88)	1.54 (0.79-3.02)	–
**Dense**					
Non-symptomatic	11 of 117	1.00	1.00	1.00	–
Symptomatic	21 of 97	2.63 (1.26-5.47)	1.71 (0.75-3.90)	1.63 (0.68-3.91)	–

HR^1^: Crude.

HR^2^: Adjusted for diagnostic age (continuous), tumour size (mm), ALNI, grade, ER, PgR and diagnostic period.

HR^3^ Adjusted for same factors as HR^2^ but also for BMI at baseline (continuous), HRT at diagnosis and diagnostic period.

HR^4^: Adjusted for the same factors as HR^3^ but also for breast density.

*Freedmans% express to what extent covariates can explain the differences in mortality between non-symptomatic vs. symptomatic patients.

In the unadjusted model, symptomatic cases had a higher risk of breast cancer death as compared to non-symptomatic regardless of breast density, Table 5. The HRs were attenuated in the adjusted models, and did not reach statistical significance. Estimated by Freedmans%, diagnostic age, tumour size, ALNI, grade, ER and PgR explained 55.6% of the observed differences in mortality between non-symptomatic and symptomatic cases. Additional adjustment for breast density caused only a minor change in Freedmans%, +0.6 per cent units.

All results remained similar excluding women with distant metastases at diagnosis (data not shown). Women with dense, as compared to fatty breasts, had a decreased risk of death from causes other than breast cancer in the crude model including all subjects, HR: 0.42:0.20-0.91, p-value for trend 0.03. The HR was 0.68(0.31-1.49) adjusted for age at diagnosis and diagnostic period, and 0.74(0.31-1.73) adjusted for age, BMI, HRT and diagnostic period (Additional file 1: Table S1 and Table S2). Results were similar after adjustment for planned chemotherapy and planned anti-hormonal therapy. Adjustment for radio-therapy caused only minor changes in the results but diminished the hazard ratios slightly. In the fully adjusted models in Table 4, HR^3^ changed from 2.59:1.08-6.20 to 2.51:1.04-6.02 and HR^4^ changed from 2.66:1.11-6.38 to 2.59:1.08-6.23 for women with dense breasts. In the fully adjusted models in Table 5 HR^3^ changed from 1.55:0.95-2.52 to 1.44:0.87-2.38 and HR^4^ from 1.54:0.94-2.52 to 1.45:0.88-2.40 for women with symptomatic tumours.

## Discussion

In the present study high breast density was positively associated with death from breast cancer, in a dose–response pattern. Moreover, this association was most pronounced among symptomatic women. Breast density did not substantially contribute to the difference in survival between women with non-symptomatic vs. symptomatic cancers.

Some methodological issues have to be considered. All Swedish residents are given a unique civil registration number at birth which facilitates record-linkage. The Swedish Cause of Death Registry offers complete data with a coverage of 97.3% in 2008 [32] and the cause of death for malignant tumours has been shown to be correct in 90% of cases [33]. Hence, information on cause of death in the present study is expected to have a high completeness and correctness.

Breast density in this study was estimated qualitatively by several radiologists. Both qualitative and quantitative methods of measuring mammographic density have shown an association between density and breast cancer risk [1]. Quantitative measurements are thought to be more exact and reliable [34]. However, there is no consensus which quantitative measure of breast density to use [35], which is why we consider it relevant to use a readily available qualitative mode of assessment such as a modified BI-RADS. Known determinants for breast density (BMI, HRT, age and menopausal status) were distributed as expected in relation to different categories of density in this study, indicating a valid measurement of breast density. No formal assessment of inter-observer variability was performed at the initial estimation of breast density in this study, which is a limitation. However, in a not yet published study, 1200 recent screening mammograms were prospectively double-read by the same observers as in the present investigation. When applying the BIRADS classification with the modification described above, a kappa coefficient of 0.60 (0.55-0.65, 95% confidence interval) and a quadratic weighted kappa of 0.66 (0.62-0.70, 95% confidence interval) were found. This provides support of a substantial inter-observer agreement between the radiologists.

There was a change from analogue to digital mammography at our institution in 2004. Difference in acquisition method has been reported not to be of great importance when using a qualitative density measure such as BI-RADS [35].

BMI was used for adjustment as a potential confounder of the possibility to detect a tumour clinically or at screening mammography. It would have been more accurate to adjust for BMI at diagnosis but this information was not available. Weight changes over time cannot be excluded, which could have resulted in residual confounding. Weight gain and a consequent rise in BMI is probably the most likely change to occur with increasing age [36,37]. We believe this non-differential misclassification could have attenuated the effect seen among women with fatty breasts.

Women with dense breasts, participating in the general screening program in Malmö, were invited at 18 instead of 24 months intervals. This may explain why the interval cancers were only slightly more common among women with dense breasts, which would otherwise have been expected according to data from previous studies [38,39]. This weak association between interval cancers and high breast density could also have attenuated a potential adverse effect of density on survival in women with non-symptomatic cancers. Indeed, the strongest effect of density was seen in symptomatic cases. Interval cancers, which might have been missed at screening due to a masking effect at mammography, were included in this group, although the results may also indicate an independent effect of density on survival. There is however a problem with small numbers of breast cancer deaths in the stratified analyses, resulting in wide confidence intervals and consequently poor precision, why the results must be interpreted with caution. However, our main result is not a difference between symptomatic and non-symptomatic women but instead an over-all association between breast density and survival.

The increased mortality seen among women with dense breasts could be explained by competing mortality from other causes than breast cancer in women with fatty breasts. This seems unlikely however, as fewer women died from other causes than from breast cancer, and women who died from other causes died at a higher age. Moreover, although fatty breasts were associated with an increased risk of death from other causes, after adjustments for age, BMI, HRT and diagnostic period, these results were considerably attenuated and not statistically significant. It is also necessary to consider potential effects of lead time but to completely adjust for lead time bias is a challenging task. We adjusted our results for age, tumour size, axillary lymph node involvement, grade, hormone-receptor status, use of HRT and BMI. All this factors are likely to be associated with lead time and we believe these adjustments would have diminished potential effects of lead time bias. We are however aware that there could be residual confounding regarding lead time, which may have influenced our results through earlier tumour detected in women with fatty breasts. If so, this would then have led to a spuriously better survival in women with fatty breasts.

It is well known that younger women with breast cancer tend to have more aggressive tumours and younger women tend to have denser breasts. However, the proportion of younger women in this cohort is relatively low; mean age at diagnosis was 64.4 years (SD 7.5). All analysis were adjusted for age, tumour size, axillary lymph node involvement, grade and hormone-receptor status, which we believe would have diminished potential confounding by more aggressive tumours in younger women.

Results from previous studies on breast density and breast cancer specific survival are difficult to compare due to differences in methodology. The present and three previous studies [4,5,14] used different qualitative classifications of breast density. Two studies used quantitative estimation of density by a computer-assisted method but one of these studies included only 27 breast cancer deaths [17]. In a recent study from Sweden, evaluating breast density quantitatively, comparing 1115 screening-detected breast cancers and 285 interval cancers in postmenopausal women, women with interval cancers had generally more aggressive tumour characteristics and worse 5 year survival, although these differences were most pronounced among women with non-dense breasts [40]. There are however several differences between their and our study. Firstly, the study by Eriksson and al. used quantitative, computer assisted measures of breast density while we estimated breast density qualitatively. Secondly, symptomatic women not diagnosed as interval cancers were excluded from the study by Eriksson et al. It was not possible to study interval cancer separately in our analysis due to small numbers, which would indeed have been interesting. Thirdly, the end point in the study by Eriksson et al. was 5 year breast cancer specific survival, while most women in our study had a considerably longer follow-up, median 7.8 years (0.5-19.1). The limited number of events in our study must also be considered although the number of events was not given in the study by Eriksson et al.

Two investigations both used the BI-RADS classification of breast density in four categories [15,16] but differed in size and design. Moreover, Chui et al. [5] estimated breast density at baseline in a screening program instead of at diagnosis, which may to some extent have attenuated their findings given potential changes in breast density over time. The results by Chiu et al., also based on a Swedish population, were similar to ours with an increased risk of breast cancer death in relation to density and with an even more increased risk in adjusted analyses. A problem in the majority of previous, and the present study, was the low number of included events, ranging from 26 [14] to 127 [5]. Despite a mean follow-up time of 7.8 years, the present study only included 76 events in the main analysis. On the other hand, the study by Gierach et al., based on data from the US Breast Cancer Surveillance Consortium, included 9232 women out of whom 889 died from breast cancer [16]. Their results showed no association between breast density and death from breast cancer. In that study nearly fifteen percent of women were treated with breast conserving surgery and not treated with radiotherapy, which most women in Sweden would have been. Women with BI-RADS category 1 were also to a lesser extent treated with chemotherapy than women in BI-RADS category 4, despite women with fatty breasts having more adverse tumour characteristics. Their results were indeed stratified for stage and adjusted for therapy but it could be that other population related differences, e.g. lack of population based mammography screening in the United States and differences regarding health care financing make results difficult to compare.

The mechanism underlying the association between breast density and breast cancer is not clear. Several studies have addressed the issue of an association between breast density and prognostic tumour characteristics but in most of these studies, no association or conflicting results have been seen between density and tumour size, ALNI or hormone receptor status [41]. Genetic factors could be of importance and there is evidence of common genetic polymorphisms related to both breast density and breast cancer development [42]. Apart from underlying genetics, breast density could biologically reflect hormonal factors [12,43,44], and factors related to oxidative stress [45,46], which could affect epithelial and/or stroma related processes [45-49]. It is possible to hypothesise that such factors could also influence breast cancer risk and/or outcome.

## Conclusions

In conclusion, in this study, high breast density at diagnosis may be associated with a decreased breast cancer specific survival. This association appears to be stronger in women with symptomatic cancers.

## Abbreviations

HRT: Hormone replacement therapy; BMI: Body mass index; MDCS: Malmö diet and cancer study; SD: Standard deviation; ALNI: Axillary lymph node involvement; ER: Estrogene receptor; PgR: Progesterone receptor; BIRADS: Breast imaging reporting and data system; ANOVA: Analysis of variance; HR: Hazard ratio.

## Competing interests

All of the authors declare no financial or nonfinancial competing interests.

## Authors’ contributions

ÅO contributed in data acquisition, analysed and interpreted the data and drafted the article. HS contributed in data acquisition on breast density, conception, design, analysis and interpretation of data and critically revised the article. SB performed tissue micro arrays and critically revised the article. SZ contributed in data acquisition on breast density, conception, design, analysis and interpretation of data and also critically revised the article. JM contributed to conception and design, analysis and interpretation of data and critically revised and drafted the article. All authors read and approved the final manuscript.

## Pre-publication history

The pre-publication history for this paper can be accessed here:

http://www.biomedcentral.com/1471-2407/14/229/prepub

## Supplementary Material

Additional file 1: Table S1 Breast density and subsequent death from other cause than BC in relation to mode of detection. **Table S2.** Mode of detection in relation to death from other cause than BC and breast density. Click here for file
