# Supplementary material for: Breast density and mode of detection in relation to breast cancer specific survival: a cohort study
Source: BMC Cancer. 2014 Mar 28;14:229. doi: 10.1186/1471-2407-14-229 (PMC3986605; doi:10.1186/1471-2407-14-229)
Supplement: Additional file 1: Table S1 — Breast density and subsequent death from other cause than BC in relation to mode of detection. Table S2. Mode of detection in relation to death from other cause than BC and breast density. [file 1471-2407-14-229-S1.doc]

Table S1**.** Breast density and subsequent death from other cause than BC in relation to mode of detection.

| Mode of detection | Breast density | Rate of deaths other cause (n=47/619) | HR^1^ | HR^2^ | HR^3^ | HR^4^ | HR^5^ |  |
| --- | --- | --- | --- | --- | --- | --- | --- | --- |
| All | Fatty | 13/93 | 1.00 | 1.00 | 1.00 | 1.00 | 1.00 |  |
|  | Moderately | 21/312 | 0.58(0.29-1.16) | 0.68(0.34-1.38) | 0.71(0.35-1.44) | 0.70(0.34-1.46) | 0.72(0.35-1.50) |  |
|  | Dense | 13/214 | 0.42(0.20-0.91) | 0.68(0.31-1.49) | 0.73(0.32-1.68) | 0.74(0.31-1.73) | 0.74(0.32-1.75) |  |
| *p-value for trend* |  |  | *0.032* | *0.353* | *0.478* | *0.498* | *0.516* |  |
| Non-symptomatic | Fatty | 6/50 | 1.00 | 1.00 | 1.00 | 1.00 | -- |  |
|  | Moderately | 9/183 | 0.48(0.17-1.34) | 0.56(0.20-1.58) | 0.63(0.22-1.82) | 0.66(0.22-1.97) | -- |  |
|  | Dense | 7/117 | 0.46(0.15-1.36) | 0.74(0.24-2.31) | 0.91(0.28-2.92) | 0.96(0.27-3.38) | -- |  |
| *p-value for trend* |  |  | *0.210* | *0.665* | *0.917* | *0.989* |  |  |
| Symptomatic | Fatty | 7/43 | 1.00 | 1.00 | 1.00 | 1.00 | -- |  |
|  | Moderately | 12/129 | 0.73(0.29-1.86) | 0.84(0.32-2.18) | 0.81(0.30-2.16) | 0.86(0.32-2.33) | -- |  |
|  | Dense | 6/97 | 0.40(0.13-1.19) | 0.62(0.20-1.90) | 0.58(0.18-1.92) | 0.59(0.18-1.97) | -- |  |
| *p-value for trend* |  |  | *0.092* | *0.401* | *0.374* | *0.390* |  |  |

HR^1^ Crude, HR^2^ Adjusted for diagnostic age and diagnostic period, HR^3^ Adjusted as HR^2^ and in addition for BMI (baseline), HR^4^ Adjusted as HR^3^ and in addition for HRT at diagnosis, HR^5^ Adjusted as HR^4^ and in addition for detection mode (non-symptomatic or symptomatic)

Table S2. Mode of detection in relation to death from other cause and breast density.

| Breast density | Mode of detection | Rate of  deaths other cause  (n=47/619) | HR^1^ | HR^2^ | HR^3^ | HR^4^ | HR^5^ |  |
| --- | --- | --- | --- | --- | --- | --- | --- | --- |
| All | Non-symptomatic | 22/350 | 1.00 | 1.00 | 1.00 | 1.00 | 1.00 |  |
|  | Symptomatic | 25/269 | 1.69(0.95-2.99) | 1.42(0.79-2.56) | 1.51(0.83-2.74) | 1.53(0.84-2.79) | 1.51(0.82-2.76) |  |
| *Freedman%** |  |  |  | *33.2* | *21.5* | *18.9* | *21.5* |  |
| Fatty/moderately dense | Non-symptomatic | 15/233 | 1.00 | 1.00 | 1.00 | 1.00 |  |  |
|  | Symptomatic | 19/172 | 1.91(0.97-3.76) | 1.56(0.78-3.14) | 1.65(0.82-3.33) | 1.66(0.82-3.35) |  |  |
|  |  |  |  |  |  |  |  |  |
| Dense | Non-symptomatic | 7/117 | 1.00 | 1.00 | 1.00 | 1.00 |  |  |
|  | Symptomatic | 6/97 | 1.21(0.41-3.61) | 1.13(0.37-3.42) | 1.07(0.34-3.35) | 1.16(0.36-3.67) |  |  |
|  |  |  |  |  |  |  |  |  |

HR^1^ Crude, HR^2^ Adjusted for diagnostic age and diagnostic period, HR^3^ Adjusted as HR^2^ and in addition for BMI (baseline), HR^4^ Adjusted as HR^3^ and in addition for HRT at diagnosis, HR^5^ Adjusted as HR^4^ and in addition for breast density

* Freedmans % (in italics) express to what extent co-variates can explain the differences in mortality between screening vs. not screening detected cases.
